# Culture density influences the functional phenotype of human macrophages

**DOI:** 10.3389/fimmu.2023.1078591

**Published:** 2023-03-10

**Authors:** Adele V. Ruder, Lieve Temmerman, Joep M.A. van Dommelen, Jan Nagenborg, Chang Lu, Judith C. Sluimer, Pieter Goossens, Erik A.L. Biessen

**Affiliations:** ^1^ Cardiovascular Research Institute Maastricht (CARIM), Department of Pathology, Maastricht University Medical Center (UMC), Maastricht, Netherlands; ^2^ BHF Centre for Cardiovascular Science, University of Edinburgh, Edinburgh, United Kingdom; ^3^ Institute for Molecular Cardiovascular Research, RWTH Aachen University, Aachen, Germany

**Keywords:** macrophage, quorum sensing, culture density, *in vitro*, macrophage function

## Abstract

Macrophages (MΦ) are commonly cultured *in vitro* as a model of their biology and functions in tissues. Recent evidence suggests MΦ to engage in quorum sensing, adapting their functions in response to cues about the proximity of neighboring cells. However, culture density is frequently overlooked in the standardization of culture protocols as well as the interpretation of results obtained *in vitro*. In this study, we investigated how the functional phenotype of MΦ was influenced by culture density. We assessed 10 core functions of human MΦ derived from the THP-1 cell line as well as primary monocyte-derived MΦ. THP-1 MΦ showed increasing phagocytic activity and proliferation with increasing density but decreasing lipid uptake, inflammasome activation, mitochondrial stress, and secretion of cytokines IL-10, IL-6, IL-1β, IL-8, and TNF-α. For THP-1 MΦ, the functional profile displayed a consistent trajectory with increasing density when exceeding a threshold (of 0.2 x 10^3^ cells/mm^2^), as visualized by principal component analysis. Culture density was also found to affect monocyte-derived MΦ, with functional implications that were distinct from those observed in THP-1 MΦ, suggesting particular relevance of density effects for cell lines. With increasing density, monocyte-derived MΦ exhibited progressively increased phagocytosis, increased inflammasome activation, and decreased mitochondrial stress, whereas lipid uptake was unaffected. These different findings in THP-1 MΦ and monocyte-derived MΦ could be attributed to the colony-forming growth pattern of THP-1 MΦ. At the lowest density, the distance to the closest neighboring cells showed greater influence on THP-1 MΦ than monocyte-derived MΦ. In addition, functional differences between monocyte-derived MΦ from different donors could at least partly be attributed to differences in culture density. Our findings demonstrate the importance of culture density for MΦ function and demand for awareness of culture density when conducting and interpreting *in vitro* experiments.

## 1 Introduction

Macrophages (MΦ) have attracted growing interest as a therapeutic target due to their dynamic role in various pathologies, including inflammatory diseases and cancer (1–3). Cell lines like THP-1, J774 or RAW264.7, or primary MΦ derived from bone marrow cells or blood monocytes are commonly used as models of human MΦ in biological assays or screening tools *in vitro* (1). To ensure reproducibility and comparability of results obtained from MΦ cultured *in vitro*, standardized cultivation protocols optimized for each cell type and assay are essential (4, 5). However, culture density at point of observation is an often-overlooked factor and either not specified at all or insufficiently substantiated by experimental data. Even though seeding density is usually mentioned and cells are often seeded at similar densities, differences in growing protocols, experimenters, and experimental conditions may result in density differences during experimentation. This is particularly worrisome in view of the reported importance of local contact and context for MΦ viability (6) and recent growing evidence of quorum sensing by MΦ (7).

Quorum sensing, a term adopted from bacteriology, refers to the modulation of gene expression by diffusible molecular cues (autoinducers) which convey information about density between cells (8–10). Quorum sensing had originally been established for biofilm formation of bacteria (8) but has recently also been observed in MΦ (7, 9, 11). In RAW264.7 MΦ, gelsolin was identified as autoinducer of programmed cell death 4 (PDCD4) expression which increased at higher cell density (11). Moreover, a quorum sensing mechanism has been observed in mice where the inflammation-resolving effect of nitric oxide (NO) was found to be dependent on the density of NO-producing MΦ (7).

Along with quorum sensing, cell density is vital for MΦ proliferation and viability. Indeed, MΦ were found to resume exponential growth faster (12), and to exhibit a more mature phenotype (13) and improved viability (12) at lower density. At higher density on the other hand, more MΦ adopted a high activation state (9). Moreover, culture density has been shown to influence several key functions of MΦ, including cytokine secretion (9, 13), polarization (13), phagocytosis (14), accumulation of esterified cholesterol (15), formation of multinucleated giant cells (16), and inhibition of mycobacterial growth (17). However, previous studies mostly compared two densities (“high” versus “low”) for only a single function, and either focused on murine MΦ cell lines or on bone marrow-derived MΦ (BMDM). So far, a comprehensive study of the functional implications of density in human MΦ models is lacking. Failure to account for density differences may introduce bias as it remains unclear whether observed effects of a treatment agent are merely attributable to its effect on culture density, either by promoting proliferation and cell survival, or by inducing cell death.

The aim of this study was to investigate the influence of culture density on human MΦ function. Using high-throughput measurement of 10 functional parameters, we studied the phenotype of THP-1 MΦ as well as human primary monocyte-derived MΦ (MDM). Moreover, we considered the colony-forming growth pattern of THP-1 cells, and donor-specific differences in MDM as additional modulators of culture density-related functional changes.

## 2 Materials and methods

### 2.1 THP-1 cell culture and differentiation to MΦ

THP-1 cells were seeded at increasing densities in 96-well black clear-bottom imaging microplates (Corning #353219) in RPMI medium with HEPES and GlutaMAX (Gibco #72400-021) supplemented with 10% heat-inactivated (30 minutes at 56°C) fetal bovine serum (FBS; SERANA #S-FBS-SA-015) and 1% penicillin-streptomycin (Gibco #15070-063). Seeding densities were 5,000, 12,000, 21,000, 37,000, 53,000, 69,000, or 87,000 cells/well, denoted as x 10^3^ cells/mm^2^ (0.16, 0.38, 0.66, 1.16, 1.66, 2.16, or 2.72 x 10^3^ cells/mm^2^, respectively) that were rounded to one decimal digit to improve readability (0.2, 0.4, 0.7, 1.2, 1.7, 2.2, or 2.7 x 10^3^ cells/mm^2^, respectively). THP-1 cells were differentiated into MΦ by exposure to 2000 nM phorbol 12-myristate 13-acetate (PMA; Sigma #P1585) for 48 hours at 37°C, 5% CO_2_ after which they were rested for 24 hours in fresh culture medium before performing the functional assays.

### 2.2 PBMC isolation, monocyte isolation, and differentiation to MΦ

Peripheral blood mononuclear cells (PBMCs) were isolated from leukocyte reduction system cones, a by-product of thrombopheresis of routine blood donations from healthy volunteers collected at the University Hospital RWTH Aachen, Germany, by density centrifugation with Lymphoprep™ (STEMCELL Technologies #07861). Isolated PBMCs were cryopreserved for later use in one-third freezing medium containing 75% FBS and 25% dimethyl sulfoxide (DMSO; Merck #102950). CD14 MicroBeads (Miltenyi #130-096-052) were used to positively select CD14+ monocytes, following the manufacturer’s protocol. Monocytes of 6 donors were pooled before seeding at the respective densities in 96-well black clear-bottom imaging microplates, or kept separate and seeded in 384-well plates (Greiner Bio-One #781866) in culture medium containing RPMI medium with HEPES and GlutaMAX supplemented with 10% heat-inactivated FBS and 1% penicillin-streptomycin. Seeding densities were 30,000, 64,000, 98,000, 132,000, 166,000, or 200,000 cells/well, denoted as x 10^3^ cells/mm^2^ (0.94, 2.0, 3.06, 4.13, 5.19, or 6.25 x 10^3^ cells/mm^2^, respectively) that were rounded to integers to improve readability (1, 2, 3, 4, 5, or 6 x 10^3^ cells/mm^2^, respectively). At 384-well format, cells were plated at 13,000 cells/well. Monocytes were differentiated into MΦ with 100 ng/ml recombinant human macrophage colony-stimulating factor (M-CSF) (ImmunoTools #11343113) for 7 days at 37°C, 5% CO_2_ with one medium change.

### 2.3 Functional high-throughput measurements

MΦ functions were assessed using the “MacroScreen” high-content analysis (HCA) platform developed in-house, a semi-automated microscale (96- to 384-well format) assay platform to perform an expanding range of fluorescence-based functional assays (18). All MacroScreen assays have been benchmarked against conventional mesoscale assays. Images were taken using the BD Pathway 855 automated fluorescent microscope (BD Biosciences) by taking 9 images per well with a 10x Olympus 0.40 NA objective (96-well format) or 20x Olympus 0.75 NA objective (384-well format). All experiments were performed in n=3-6 replicates. Images were analyzed with CellProfiler software version 4.0.4 (19) by creating a digital segmentation mask for each cell based on its nuclear staining signal using the IdentifyPrimaryObjects module followed by the ExpandOrShrinkObject module. Percentage of positive cells was measured using the MeasureObjectIntensity module followed by the ClassifyObjects module, which determines the amount of positive cells relative to the total number of segmented objects in the image. Distance to the closest neighboring cell was measured per image, for each cell, using the MeasureObjectNeighbors module in CellProfiler 4.0.4 (19).

### 2.4 Phagocytosis

Cells were incubated with 25 μl/ml pHrodo™ Red Zymosan Bioparticles™ (ThermoFisher Scientific #P35364) per well in culture medium for 1 hour at 37°C, 5% CO_2_. Zymosan particles taken up by cells are reflected by tetramethyl rhodamine isothicyanate (TRITC) fluorescence signal. Nuclei were stained with Hoechst 33342 (Sigma #B2261) in culture medium for 10 minutes at 37°C, 5% CO_2_ which was replaced by PBS before imaging.

### 2.5 Lipid uptake

Low-density lipoprotein (LDL) was isolated from serum of healthy volunteers *via* density centrifugation and oxidized using CuSO_4_ as described previously (20). Cells were incubated with 8 μg/ml oxidized LDL (oxLDL) pre-mixed with 2 μg/ml TopFluor^®^ Cholesterol (Avanti Polar Lipid #810255P) in culture medium for 3 hours at 37°C, 5% CO_2_, reflected by fluorescein isothiocyanate (FITC) fluorescence signal. Nuclei were stained with Hoechst 33342 in culture medium for 10 minutes at 37°C, 5% CO_2_ which was replaced by PBS before imaging.

### 2.6 Inflammasome

Cells were primed with 50 ng/ml LPS from E. coli (Invivogen #tlrl-eblps) for 3 hours at 37°C, 5% CO_2_. 10 μM nigericin (Invivogen #tlrl-nig) for 1 hour at 37°C, 5% CO_2_ served as second signal for inflammasome activation. Fc receptor was blocked with Fc receptor binding inhibitor antibody (Invitrogen #14-9161-73) before cells were fixed using 2% PFA with 5 mM EDTA in PBS and permeabilized using 5% FBS and 0.5% Triton X-100 in PBS for 20 minutes on ice. Next, the intracellular adapter protein apoptosis associated speck-like protein containing a CARD (ASC) was stained using PE-conjugated anti-ASC antibody (clone HASC-71; BioLegend #653904) overnight at 4°C. Nuclei were stained with Hoechst 33342 in PBS for 10 minutes on ice, and cells were washed with and imaged in PBS afterwards.

### 2.7 Mitochondrial stress

Mitochondrial stress was induced using 1200 nM staurosporine (Sigma #S4400) for 1 hour at 37°C, 5% CO_2_. Mitochondria and nuclei were stained simultaneously with 250 nM MitoTracker™ Deep Red FM (ThermoFisher Scientific #M22426) and Hoechst 33342, respectively, in culture medium for 30 minutes at 37°C, 5% CO_2_ before imaging in PBS. Alexa 594 fluorescence signal reflects mitochondrial staining dependent on membrane potential.

### 2.8 Proliferation

Cells were incubated with 10 μM 5’ethynyl-2’-deoxyuridine (EdU; ThermoFisher Scientific #A10044) for 2 hours at 37°C, 5% CO_2_. Next, cells were fixated with 3.7% PFA in PBS for 15 minutes and washed twice with PBS before permeabilization with 0.1% Triton X-100 in PBS for 15 minutes. After another wash with PBS, Click-iT reaction cocktail (ThermoFisher Scientific #C10269) including Alexa Fluor 594 azide (ThermoFisher Scientific, #A10270) prepared according to the manufacturer’s instructions was added. After 30 minutes, cells were washed with PBS and nuclei stained with Hoechst 33342 for 15 minutes. Cells were imaged in 1:5 KI quencher (1M KI in 10 mM KH_2_PO_4_) in PBS.

### 2.9 Multiplex ELISA

THP-1-derived MΦ were stimulated with 50 ng/ml LPS from E. coli for 6 hours at 37°C, 5% CO_2_. Supernatant was collected and cytokine levels of IL-1β, IL-6, IL-8, IL-10, IL-12p70, and TNF-α were measured in a custom V-plex human cytokine ELISA (MSD Meso Scale Diagnostics) according to the manufacturer’s protocol. Measured levels of IL-12p70 were close to or below the standard curve (0.1169 pg/ml), thus these data are not presented here.

### 2.10 Dimensionality reduction and visualization

We calculated the mean of the replicates for each feature of HCA functional data and cytokine data at the same density, resulting in a density-feature matrix with 7 rows (densities) and 10 columns (features/functions). The functional profile of THP-1 MΦ for the 7 different densities was analyzed in an integrated manner by two-dimensional Principal Component Analysis (PCA), where the relative contribution of each individual function to the first (PC1) and second principal component (PC2) axes was visualized outside the X and Y axes.

### 2.11 Statistical analysis

Data are expressed as mean ± SEM, unless stated otherwise. Normal distribution was assessed by Shapiro-Wilkes normality test, and equality of variances by Brown-Forsythe test. For normally distributed data with equal variance, significance was assessed by one-way ANOVA followed by Tukey’s multiple comparisons test at a significance level of p <0.05. For normally distributed data with unequal variance, significance was assessed by Brown-Forsythe ANOVA and Dunnett’s T3 multiple comparisons test, and Kruskal-Wallis test with Dunn’s multiple comparisons test was used for not normally distributed data. All statistical analyses were performed using GraphPad Prism 8 software.

## 3 Results

### 3.1 Density influences the functional phenotype of MΦ

**Figure 1:**
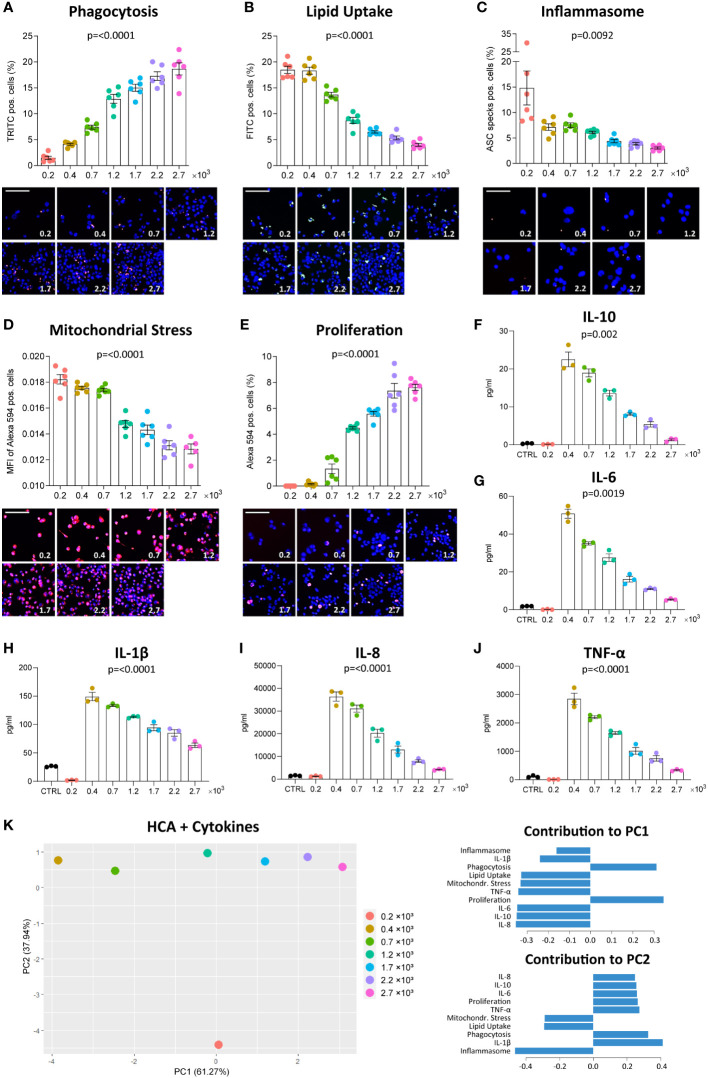
Density affects MΦ function. THP-1 cells were seeded at the respective densities (0.2-2.7 x 10^3^ cells/mm²) and differentiated with 2000 nM PMA for 48 hours followed by a resting period of 24 hours. Phagocytosis of zymosan-coated beads **(A**, red**)**, uptake of fluorescently-labelled oxLDL **(B**, green**)**, inflammasome activation **(C**, red**)**, mitochondrial stress in response to staurosporine **(D**, red**)** and EdU incorporation in proliferating cells **(E**, red**)** were assessed using the fluorescent imaging MacroScreen platform. Hoechst was used for nuclear labelling (blue). In the representative fluorescent images, the scale bar refers to 50 μM. In the representative images for the inflammasome assay, the scale bar refers to 25 μM. Secretion of IL-10 **(F)**, IL-6 **(G)**, IL-1β **(H)**, IL-8 **(I)**, and TNF-α **(J)** in response to 6-hour stimulation with 50 ng/ml LPS were measured by multiplex ELISA. Cells in the control (CTRL) condition were seeded at 1.7 x 10^3^ cells/mm² and not stimulated with LPS. The indicated p-values refer to overall significance, p-values of all group comparisons can be found in **Supplementary Table 1**. **(K)** PCA plot of the high-content analysis (HCA) data and cytokine measurements with the factor loadings for PC1 and PC2. MFI: Mean fluorescence intensity.

We assessed the functional phenotype of THP-1-derived MΦ seeded at 7 different densities ranging from 0.2 to 2.7 x 10^3^ cells/mm^2^ using the MacroScreen platform, an HCA platform based on several fluorescent imaging-based functional assays. For clarity’s sake, we presented the p-values of all group comparisons for all assays in a significance matrix (**Supplementary Table 1**). As expected, the number of nuclei per fluorescent image increased with density in all high-content functional assays (**Supplementary Figures 1A–E**). No changes in the pH of the culture medium were observed. Whereas the phagocytic activity of THP-1 MΦ was found to increase with higher density (**Figure 1A**), uptake of oxLDL decreased (**Figure 1B**). MΦ seeded at 0.2 x 10^3^ cells/mm^2^, the lowest density in the tested density range, showed the highest inflammasome activation in response to LPS and nigericin (**Figure 1C**). Moreover, at lower densities, MΦ were found to be more resistant to staurosporine-induced mitochondrial stress (**Figure 1D**). Proliferation measured by EdU incorporation increased with density (**Figure 1E**). Secretion of the cytokines IL-10, IL-1β, IL-6, IL-8, and TNF-α in response to LPS stimulation was measured using multiplex ELISA, and was found to decrease with density, after an initial sharp increase between the two lowest densities included in the density range (0.2 and 0.4 x 10^3^ cells/mm^2^) (**Figures 1F–J**). PCA allowed separation of THP-1 MΦ seeded at different densities based on their functional profiles (**Figure 1K**). A large proportion of variation (61.27%) could be explained by PC1 which loadings included mitochondrial stress, lipid uptake, proliferation and phagocytosis, suggesting these functions to be mostly influenced by density. Variation along PC2 (largely attributable to inflammasome activation and cytokine secretion) almost exclusively manifested at the lowest density (0.2 x 10^3^ cells/mm^2^). Together, these data demonstrate the profound and variable impact of culture density on several THP-1 MΦ functions. Execution of certain functions may depend on exceeding a lower density threshold.

### 3.2 Density influences the functional phenotype of primary MΦ

**Figure 2:**
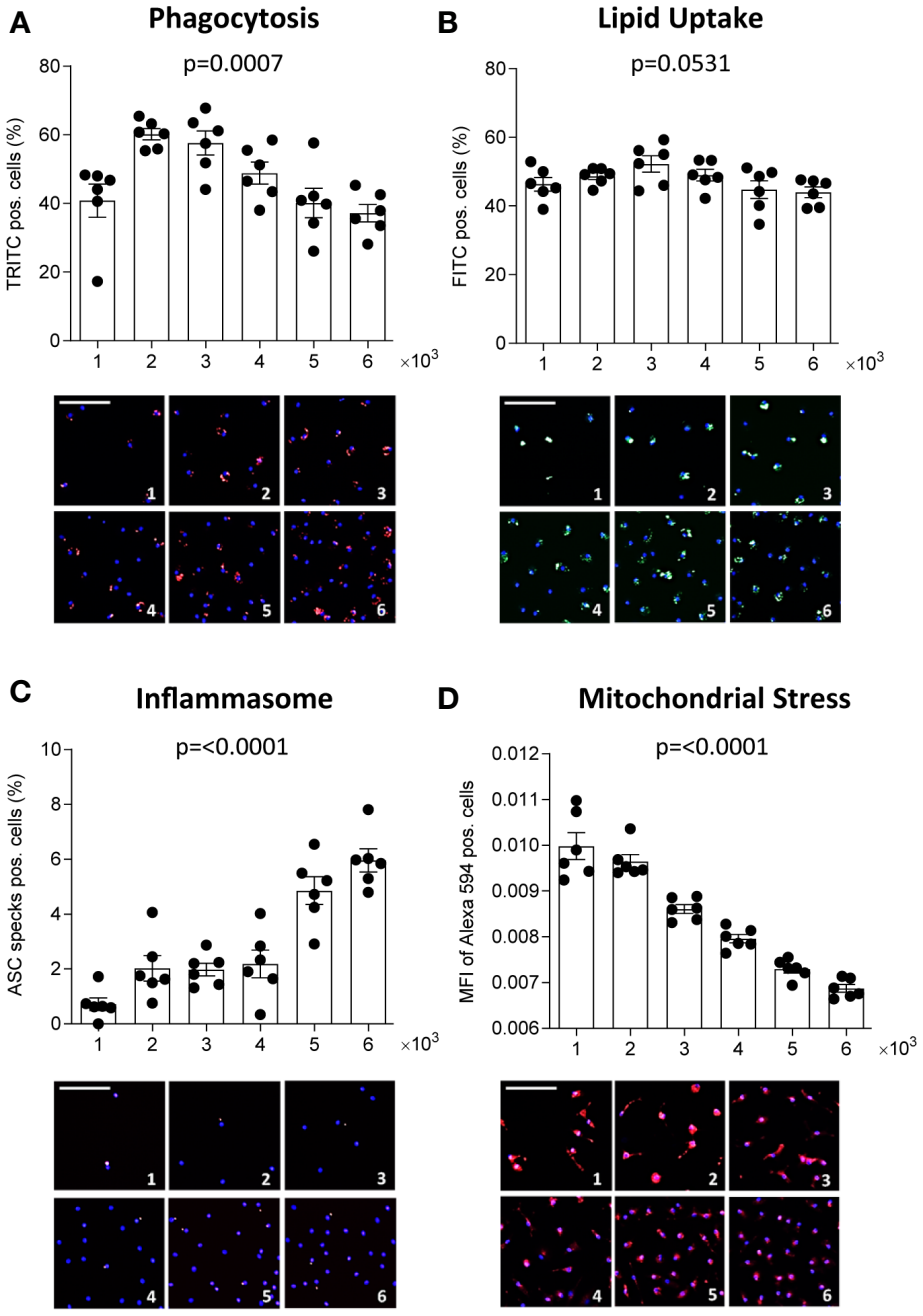
Density affects human primary MΦ function. CD14-positive monocytes were isolated from PBMCs of 6 healthy donors, pooled and seeded at the respective densities (1-6 x 10^3^ cells/mm²) and differentiated to MΦ using 100 ng/ml macrophage colony-stimulating factor (M-CSF) for 7 days. Phagocytic activity **(A**, red**)**, oxLDL uptake **(B**, green**)**, inflammasome activation **(C**, red**)**, and mitochondrial stress **(D**, red**)** were assessed using the MacroScreen platform. Hoechst was used for nuclear labelling (blue). The scale bar in the representative fluorescent images refers to 50 μM. The indicated p-values refer to overall significance, multiple comparison significance can be found in **Supplementary Table 2**. MFI: Mean fluorescence intensity.

We also investigated whether culture density influences the functional phenotype of human MDM, pooled from 6 healthy donors. A significance matrix with the p-values of all group comparisons can be found in **Supplementary Table 2**. As seen in THP-1 MΦ, the number of detected nuclei increased with increasing seeding density (**Supplementary Figures 1F–I**). Phagocytic activity increased in the lower densities but decreased again at seeding densities exceeding 3 x 10^3^ cells/mm^2^, possibly reflecting limited substrate (**Figure 2A**). Using a constant substrate-to-cell ratio, increased phagocytosis also at higher densities could be observed (**Supplementary Figure 2**). OxLDL uptake between MDM seeded at different densities was similar, contrary to THP-1 MΦ (**Figure 2B**). With increasing density, MDM demonstrated more inflammasome activation (**Figure 2C**) and were more susceptible to mitochondrial stress (**Figure 2D**). Overall, our findings show that density also affects primary MΦ and leads to changes in several functions. In MDM, these changes were less pronounced and less consistent compared to THP-1 MΦ, suggesting that the observed impact of density on MΦ function may be particularly relevant for cell lines.

### 3.3 Colony formation may contribute to density-dependent functional effects

**Figure 3:**
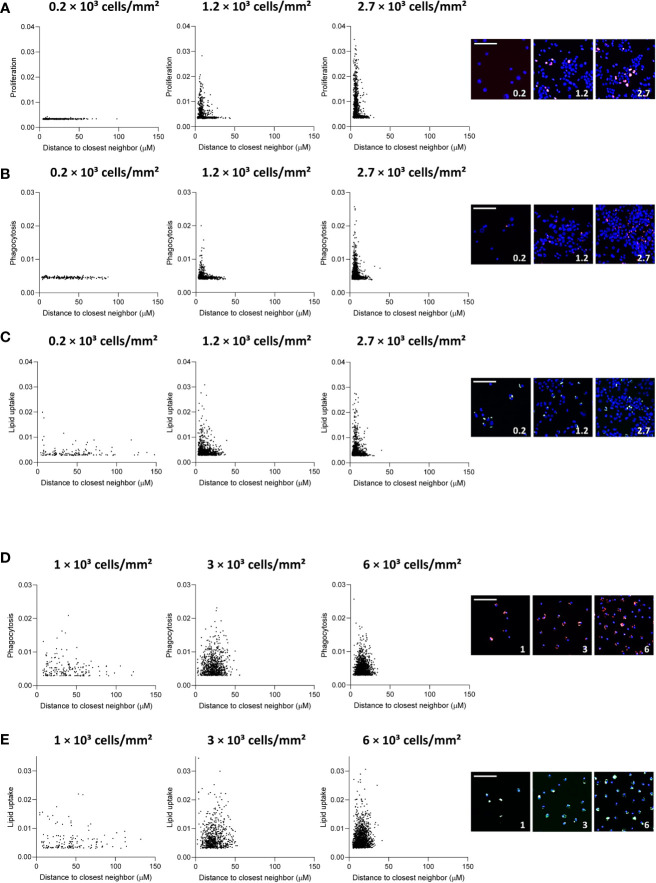
Distance to neighboring cells influences THP-1 function. Distance to closest neighbor was plotted against mean fluorescence intensity (MFI) per cell for proliferation **(A)**, phagocytosis **(B)** and lipid uptake **(C)** assays in THP-1 MΦ, and **(D)** phagocytosis and **(E)** lipid uptake assay in primary MDM, for 3 plating densities each. The scale bar corresponds to 50 μm.

We observed that THP-1 MΦ formed colonies in culture, especially if cultured at higher densities. Thus, we set out to study if at least part of the density-dependent functional changes may be attributed to changes in the colony-forming growth pattern of THP-1 cells. We assessed the association between the functional outcome of an index cell with its distance to the closest neighboring cell. Index cells that were close to neighboring cells had higher proliferation rates compared to MΦ in more scarcely populated niches (**Figure 3A**). However, at very low seeding density (0.2 x 10^3^ cells/mm^2^), MΦ proliferation was low regardless of the index cell’s distance to neighboring cells, suggesting proliferation to be linked to colony formation. Similarly, we found that at a seeding density of 0.2 x 10^3^ cells/mm^2^, MΦ with neighboring cells in close proximity had taken up fewer fluorescent beads compared to equally dense MΦ seeded at higher densities (**Figure 3B**), a finding that was less pronounced for the uptake of oxLDL (**Figure 3C**). Interestingly, a similar proximity effect on phagocytosis and lipid uptake in MDM (**Figures 3D, E**), in which we did not observe colony formation in culture, was not apparent. These findings suggest that MΦ only exert certain functions beyond a local cell density threshold.

### 3.4 Functional differences between MDM of different donors may arise from density differences

**Figure 4:**
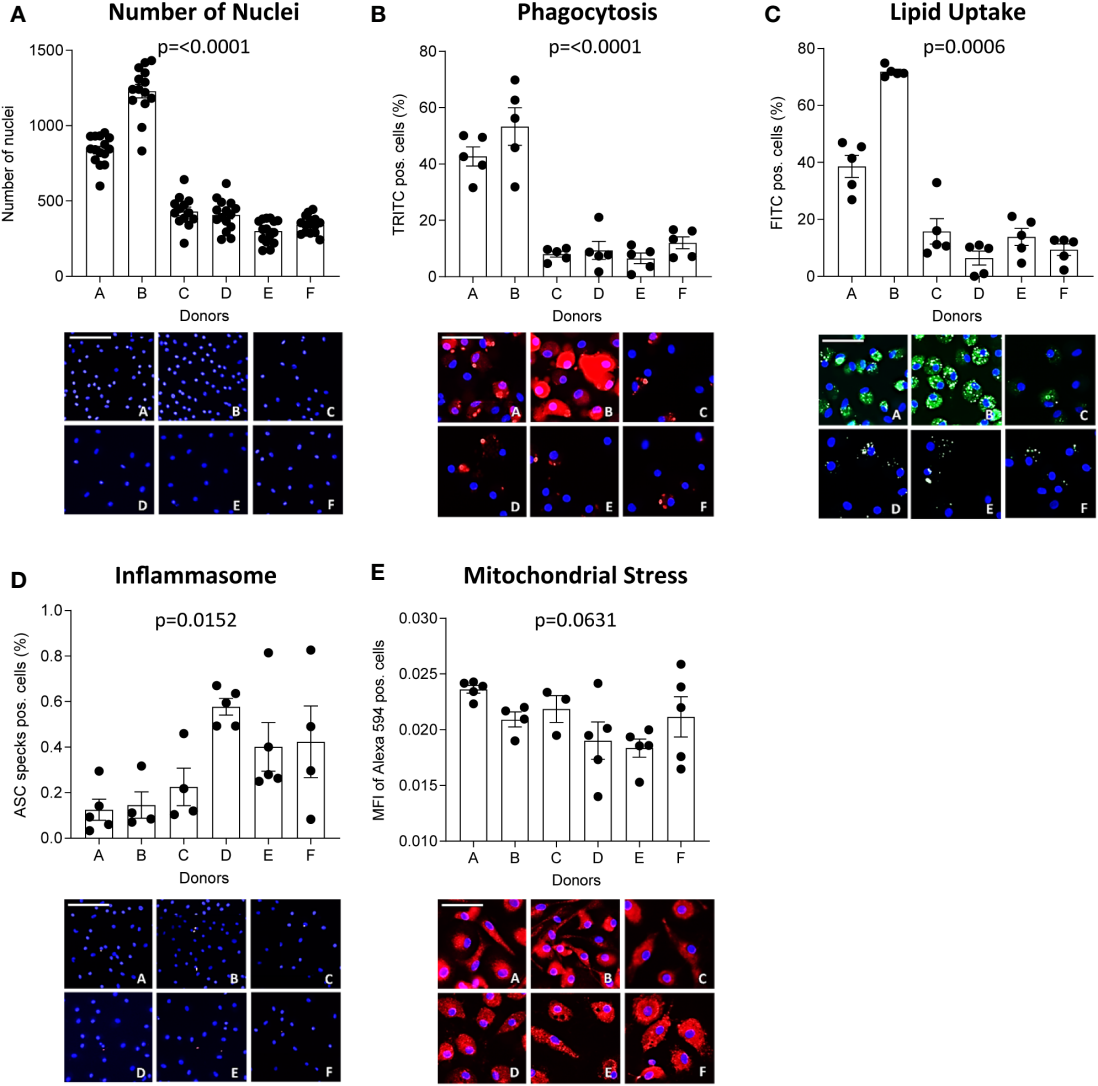
MΦ function differs between donors. CD14-positive monocytes of 6 healthy donors were seeded at 13,000 cells/well and differentiated into MΦ for 7 days using 100 ng/ml macrophage colony-stimulating factor (M-CSF). The number of nuclei per donor at experimentation **(A)** is given. Their functional phenotype was assessed using the MacroScreen platform: phagocytosis **(B**, red**)**, lipid uptake **(C**, green**)**, inflammasome activation **(D**, red**)**, and mitochondrial stress **(E**, red**)**. The scale bar corresponds to 25 μM. The indicated p-values refer to overall significance, multiple comparison significance can be found in **Supplementary Table 3**. MFI: Mean fluorescence intensity.

Inter-donor variability has previously been proposed to compromise reproducibility of *in vitro* experiments with MDM (21, 22). Therefore, we looked at functions of MDM from 6 healthy donors (A-F) plated at the same density (13,000 cells/well). A significance matrix with the p-values of all group comparisons can be found in **Supplementary Table 3**. Differences in the number of nuclei (**Figure 4A**), phagocytosis (**Figure 4B**), oxLDL uptake (**Figure 4C**), inflammasome activation (**Figure 4D**) and mitochondrial stress (**Figure 4E**) between donors were apparent. Interestingly, MDM from donors A and B, which showed higher phagocytic activity and oxLDL uptake, also had a higher number of nuclei compared to the other donors. This suggests that functional differences between MDM obtained from different donors may well be related to inter-donor differences in density, possibly resulting from differences in adherence and survival capacity.

## 4 Discussion

*In vitro* studies are an indispensable step in the screening of genes or drugs and the safety testing of compounds, before costly and time-consuming pre-clinical *in vivo* studies may commence (23). Parameters that should be considered for robust *in vitro* testing include the culture medium and supplements such as growth factors or antibiotics, the culture dish and its coating, and culture maintenance (24). In this study, we have shown that culture density, a frequently overlooked factor, is an at least equally important parameter with major impact on MΦ assay outcome. As we show, density affects the functional phenotype of both THP-1 MΦ and primary MDM, with individual functions showing distinct density responses. Moreover, our data suggest the distance of index cells to their neighbor cells to influence functionality of THP-1 MΦ but less so of MDM, and the functional profiles and density response to vary considerably between donors.

The effect of low and high culture density on individual MΦ functions has been studied previously. In BMDM, secretion of TNF-α and other cytokines (MCP-1, RANTES, MIP-1α and MIP-1β) was found to increase with cell density (9). However, another study found BMDM at higher density to secrete less pro-inflammatory cytokines, including IL-6 and TNF-α (13). These contradicting results may be explained by differences in applied seeding densities in the two studies, as these were approximately 10 times higher in the latter study. Although our study did not look at BMDM, our THP-1 MΦ results do support a decrease of cytokine secretion with increasing density. However, at the lowest density, cytokine secretion by THP-1 MΦ was at most marginal, suggesting a defective response of sparsely seeded MΦ to toll-like receptor (TLR) activation, potentially due to quiescence or compromised viability.

Although phagocytosis and lipid uptake are both clearance mechanisms, they showed a divergent density response pattern in both THP-1 MΦ and MDM. Previously, THP-1 MΦ seeded at a lower density were found to show a 5-fold higher accumulation of esterified cholesterol after exposure to acetylated LDL (acLDL), which the authors attributed to increased scavenger receptor activity and acLDL degradation (15). Moreover, murine peritoneal IC-21 MΦ showed decreased phagocytosis of latex beads when seeded at a higher density (14), which partly concurs with our findings in human primary MDM and can be attributed to the limited availability of beads per cell. Bead concentration should be increased if cells are cultured at higher density to ensure sufficient bead availability for all actively phagocytosing cells.

Primary cells or cell lines are frequently and often interchangeably used in MΦ *in vitro* studies; the latter offering the advantages of easier availability and culture maintenance, and a uniform genetic background which may increase reproducibility (21). A serious disadvantage of cell lines is that compared to primary cells, they are to a certain degree de-differentiated and show clear transcriptional and functional differences. For example, the murine J774A.1 MΦ cell line and BMDM were found to respond differently to mycobacterial infection (25). Often myeloma-derived or immortalized cell lines have aberrant contact inhibition and growth patterns (26); indeed, we found the proximity of neighboring cells to have a greater impact on THP-1 MΦ than MDM function. In THP-1 MΦ, this may be attributed to increased proliferation at closer proximity which is absent at lower density, and suggests quorum sensing to induce functional changes locally at sites of colony formation. Comparing primary human monocytes with the monocytic cell lines U-937, HL-60, and THP-1, the latter were found to most closely resemble primary monocytes (27). Nevertheless, in contrast to primary MDM, cell line-derived MΦ do not capture inter-donor variability. This might be evaded by iPSC-derived MΦ, although Vaughan-Jackson et al. recently demonstrated density-related effects also in iPSC, including a more rounded and less elongated morphology, decreased cytokine secretion, and altered surface marker expression at higher density (28). However, it could be argued that also primary MΦ in monocultures *in vitro* do not mirror the *in vivo* conditions, where MΦ are embedded in a local heterogenous tissue microenvironment with implications for cell function (21). The complex cellular and molecular microenvironment tissue MΦ are exposed to *in vivo* cannot be mimicked by exposure to a single or a few selected stimuli such as LPS and IFN-γ, or IL-4 and IL-10, as commonly done (29, 30).

Of note, we did not observe any indications for increased acidification of the culture medium which could explain the observed density effects and even at the highest density, the total cell number was most likely too low to deplete key nutrients. However, the comparability of our results on THP-1 MΦ and MDM may be impeded because different seeding densities had to be used due to the proliferating nature of the THP-1 cell line. In addition, the culturing process of MDM (7 days) and THP-1 MΦ (2 days PMA stimulation and 1 day resting) differs considerably. An extended resting period (5 days after 3 days PMA stimulation) has been shown to yield THP-1 MΦ with a phenotype closer to MDM (31). Moreover, the functional profile we provide here is not comprehensive as it only includes measurements of 5 cellular readouts and 5 cytokines. Future studies could reveal if the observed culture density effects also apply to other functions such as efferocytosis, susceptibility to apoptosis, production of reactive oxygen species (ROS), and metabolic differences.

In conclusion, our study highlights the importance of culture density for *in vitro* MΦ assay outcome and pleads for more awareness and closer monitoring of differences in cell density between conditions. This is particularly important for the THP-1 cell line which demonstrated more pronounced density effects compared to primary MDM, and when treatments are suspected to act pro-apoptotic or favor cell detachment or proliferation, which in turn impacts cell density. Moreover, a sufficiently large donor pool should be used in experiments with primary MDM to account for inter-donor variability, as the number of cells after differentiation and culture differed between donors despite seeding at the same density. Disregarding density differences may lead to secondary effects on MΦ functions, and thus misinterpretation of findings.

## Data availability statement

The raw data supporting the conclusions of this article will be made available by the authors, without undue reservation.

## Author contributions

AR, JN, LT, PG and EB contributed to the conception and design of the study. AR, JD and JN carried out the experiments. CL performed the bioinformatic analyses. PG, LT, JS and EB contributed to the interpretation of results. AR wrote the manuscript with input from all authors. All authors contributed to the article and approved the submitted version.
