# Supplementary material for: Culture density influences the functional phenotype of human macrophages
Source: Front Immunol. 2023 Mar 10;14:1078591. doi: 10.3389/fimmu.2023.1078591 (PMC10036771; doi:10.3389/fimmu.2023.1078591)
Supplement: Supplementary file 1 [file DataSheet_1.docx]

Supplementary Material

# Supplementary Figures and Tables

## Supplementary Tables

**Supplementary Table 1.** Significance matrix with p-values of group comparisons in functional assays on THP-1 macrophages. Density conditions are indicated as × 10^3^ cells/mm^2^.

| **Phagocytosis** (corresponding to Fig. 1A) | | | | | | | |  |
| --- | --- | --- | --- | --- | --- | --- | --- | --- |
| **× 10^3^** | **0.4** | | **0.7** | **1.2** | **1.7** | **2.2** | **2.7** |  |
| **0.2** | 0.1523 | | <0.0001 | <0.0001 | <0.0001 | <0.0001 | <0.0001 |  |
| **0.4** |  | | 0.0341 | <0.0001 | <0.0001 | <0.0001 | <0.0001 |  |
| **0.7** |  | |  | <0.0001 | <0.0001 | <0.0001 | <0.0001 |  |
| **1.2** |  | |  |  | 0.3623 | 0.0018 | <0.0001 |  |
| **1.7** |  | |  |  |  | 0.2933 | 0.0145 |  |
| **2.2** |  | |  |  |  |  | 0.8149 |  |
| **Lipid Uptake** (corresponding to Fig. 1B) | | | | | | | |  |
| **× 10^3^** | **0.4** | | **0.7** | **1.2** | **1.7** | **2.2** | **2.7** |  |
| **0.2** | >0.9999 | | <0.0001 | <0.0001 | <0.0001 | <0.0001 | <0.0001 |  |
| **0.4** |  | | <0.0001 | <0.0001 | <0.0001 | <0.0001 | <0.0001 |  |
| **0.7** |  | |  | <0.0001 | <0.0001 | <0.0001 | <0.0001 |  |
| **1.2** |  | |  |  | 0.0504 | 0.0006 | <0.0001 |  |
| **1.7** |  | |  |  |  | 0.6558 | 0.0183 |  |
| **2.2** |  | |  |  |  |  | 0.5003 |  |
| **Inflammasome** (corresponding to Fig. 1C) | | | | | | | |  |
| **× 10^3^** | **0.4** | | **0.7** | **1.2** | **1.7** | **2.2** | **2.7** |  |
| **0.2** | 0.5259 | | 0.5661 | 0.3857 | 0.2411 | 0.2054 | 0.163 |  |
| **0.4** |  | | >0.9999 | 0.885 | 0.0899 | 0.04 | 0.0124 |  |
| **0.7** |  | |  | 0.2864 | 0.0061 | 0.0017 | 0.0007 |  |
| **1.2** |  | |  |  | 0.0424 | 0.0014 | <0.0001 |  |
| **1.7** |  | |  |  |  | 0.9603 | 0.1122 |  |
| **2.2** |  | |  |  |  |  | 0.3429 |  |
| **Mitochondrial Stress** (corresponding to Fig. 1D) | | | | | | | |  |
| **× 10^3^** | **0.4** | | **0.7** | **1.2** | **1.7** | **2.2** | **2.7** |  |
| **0.2** | 0.6803 | | 0.4296 | <0.0001 | <0.0001 | <0.0001 | <0.0001 |  |
| **0.4** |  | | 0.9996 | <0.0001 | <0.0001 | <0.0001 | <0.0001 |  |
| **0.7** |  | |  | <0.0001 | <0.0001 | <0.0001 | <0.0001 |  |
| **1.2** |  | |  |  | 0.9255 | 0.0055 | 0.0016 |  |
| **1.7** |  | |  |  |  | 0.0864 | 0.0271 |  |
| **2.2** |  | |  |  |  |  | 0.9953 |  |
| **Proliferation** (corresponding to Fig. 1E) | | | | | | | |  |
| **× 10^3^** | **0.4** | **0.7** | | **1.2** | **1.7** | **2.2** | **2.7** |  |
| **0.2** | N/A | N/A | | N/A | N/A | N/A | N/A |  |
| **0.4** |  | 0.1936 | | <0.0001 | <0.0001 | 0.0005 | <0.0001 |  |
| **0.7** |  |  | | 0.0018 | 0.0002 | 0.0001 | <0.0001 |  |
| **1.2** |  |  | |  | 0.0123 | 0.0353 | 0.0002 |  |
| **1.7** |  |  | |  |  | 0.1985 | 0.0006 |  |
| **2.2** |  |  | |  |  |  | >0.9999 |  |
| **IL-10** (corresponding to Fig. 1F) | | | | | | | | |
| **× 10^3^** | **0.2** | | **0.4** | **0.7** | **1.2** | **1.7** | **2.2** | **2.7** |
| **CTRL** | >0.9999 | | 0.062 | 0.2215 | >0.9999 | >0.9999 | >0.9999 | >0.9999 |
| **0.2** |  | | 0.0096 | 0.0419 | 0.2625 | >0.9999 | >0.9999 | >0.9999 |
| **0.4** |  | |  | >0.9999 | >0.9999 | >0.9999 | >0.9999 | 0.3101 |
| **0.7** |  | |  |  | >0.9999 | >0.9999 | >0.9999 | 0.9146 |
| **1.2** |  | |  |  |  | >0.9999 | >0.9999 | >0.9999 |
| **1.7** |  | |  |  |  |  | >0.9999 | >0.9999 |
| **2.2** |  | |  |  |  |  |  | >0.9999 |
| **IL-6** (corresponding to Fig. 1G) | | | | | | | | |
| **× 10^3^** | **0.2** | | **0.4** | **0.7** | **1.2** | **1.7** | **2.2** | **2.7** |
| **CTRL** | >0.9999 | | 0.0509 | 0.2621 | >0.9999 | >0.9999 | >0.9999 | >0.9999 |
| **0.2** |  | | 0.0077 | 0.0509 | 0.2621 | >0.9999 | >0.9999 | >0.9999 |
| **0.4** |  | |  | >0.9999 | >0.9999 | >0.9999 | >0.9999 | 0.2621 |
| **0.7** |  | |  |  | >0.9999 | >0.9999 | >0.9999 | >0.9999 |
| **1.2** |  | |  |  |  | >0.9999 | >0.9999 | >0.9999 |
| **1.7** |  | |  |  |  |  | >0.9999 | >0.9999 |
| **2.2** |  | |  |  |  |  |  | >0.9999 |
| **IL-1β** (corresponding to Fig. 1H) | | | | | | | | |
| **× 10^3^** | **0.2** | | **0.4** | **0.7** | **1.2** | **1.7** | **2.2** | **2.7** |
| **CTRL** | 0.0117 | | <0.0001 | <0.0001 | <0.0001 | <0.0001 | <0.0001 | 0.0002 |
| **0.2** |  | | <0.0001 | <0.0001 | <0.0001 | <0.0001 | <0.0001 | <0.0001 |
| **0.4** |  | |  | 0.1783 | 0.0003 | <0.0001 | <0.0001 | <0.0001 |
| **0.7** |  | |  |  | 0.0485 | 0.0001 | <0.0001 | <0.0001 |
| **1.2** |  | |  |  |  | 0.071 | 0.0033 | <0.0001 |
| **1.7** |  | |  |  |  |  | 0.7457 | 0.0013 |
| **2.2** |  | |  |  |  |  |  | 0.0279 |
| **IL-8** (corresponding to Fig. 1I) | | | | | | | | |
| **× 10^3^** | **0.2** | | **0.4** | **0.7** | **1.2** | **1.7** | **2.2** | **2.7** |
| **CTRL** | >0.9999 | | <0.0001 | <0.0001 | <0.0001 | 0.0001 | 0.0306 | 0.7575 |
| **0.2** |  | | <0.0001 | <0.0001 | <0.0001 | <0.0001 | 0.0239 | 0.6845 |
| **0.4** |  | |  | 0.1068 | <0.0001 | <0.0001 | <0.0001 | <0.0001 |
| **0.7** |  | |  |  | 0.0003 | <0.0001 | <0.0001 | <0.0001 |
| **1.2** |  | |  |  |  | 0.0132 | <0.0001 | <0.0001 |
| **1.7** |  | |  |  |  |  | 0.1334 | 0.0023 |
| **2.2** |  | |  |  |  |  |  | 0.429 |
| **TNF-α** (corresponding to Fig. 1J) | | | | | | | | |
| **× 10^3^** | **0.2** | | **0.4** | **0.7** | **1.2** | **1.7** | **2.2** | **2.7** |
| **CTRL** | 0.9934 | | <0.0001 | <0.0001 | <0.0001 | <0.0001 | 0.0036 | 0.6971 |
| **0.2** |  | | <0.0001 | <0.0001 | <0.0001 | <0.0001 | 0.0008 | 0.2876 |
| **0.4** |  | |  | 0.0035 | <0.0001 | <0.0001 | <0.0001 | <0.0001 |
| **0.7** |  | |  |  | 0.0119 | <0.0001 | <0.0001 | <0.0001 |
| **1.2** |  | |  |  |  | 0.0043 | 0.0001 | <0.0001 |
| **1.7** |  | |  |  |  |  | 0.5181 | 0.002 |
| **2.2** |  | |  |  |  |  |  | 0.0903 |

**Supplementary Table 2.** Significance matrix with p-values of group comparisons in functional assays on primary monocyte-derived macrophages. Density conditions are indicated as × 10^3^ cells/mm^2^.

| **Phagocytosis** (corresponding to Fig. 2A) | | | | | |
| --- | --- | --- | --- | --- | --- |
| **× 10^3^** | **2** | **3** | **4** | **5** | **6** |
| **1** | 0.1624 | 0.3698 | >0.9999 | >0.9999 | >0.9999 |
| **2** |  | >0.9999 | >0.9999 | 0.0322 | 0.0061 |
| **3** |  |  | >0.9999 | 0.0848 | 0.0184 |
| **4** |  |  |  | >0.9999 | >0.9999 |
| **5** |  |  |  |  | >0.9999 |
| **Lipid Uptake** (corresponding to Fig. 2B) | | | | | |
| **× 10^3^** | **2** | **3** | **4** | **5** | **6** |
| **1** | 0.9515 | 0.2974 | 0.9248 | 0.991 | 0.9543 |
| **2** |  | 0.7995 | >0.9999 | 0.7022 | 0.5291 |
| **3** |  |  | 0.8482 | 0.1028 | 0.0563 |
| **4** |  |  |  | 0.6413 | 0.4681 |
| **5** |  |  |  |  | 0.9997 |
| **Inflammasome** (corresponding to Fig. 2C) | | | | | |
| **× 10^3^** | **2** | **3** | **4** | **5** | **6** |
| **1** | 0.2428 | 0.2771 | 0.1483 | <0.0001 | <0.0001 |
| **2** |  | >0.9999 | 0.9998 | 0.0004 | <0.0001 |
| **3** |  |  | 0.9993 | 0.0003 | <0.0001 |
| **4** |  |  |  | 0.0009 | <0.0001 |
| **5** |  |  |  |  | 0.419 |
| **Mitochondrial Stress** (corresponding to Fig. 2D) | | | | | |
| **× 10^3^** | **2** | **3** | **4** | **5** | **6** |
| **1** | >0.9999 | >0.9999 | 0.1386 | 0.004 | <0.0001 |
| **2** |  | >0.9999 | 0.2979 | 0.0113 | 0.0003 |
| **3** |  |  | >0.9999 | 0.639 | 0.0552 |
| **4** |  |  |  | >0.9999 | 0.8267 |
| **5** |  |  |  |  | >0.9999 |

**Supplementary Table 3.** Significance matrix with p-values of group comparisons in the functional assays on primary monocyte-derived macrophages of 6 different donors.

| **Number of Nuclei** (corresponding to Fig. 4A) | | | | | |
| --- | --- | --- | --- | --- | --- |
| **Donor** | **B** | **C** | **D** | **E** | **F** |
| **A** | <0.0001 | <0.0001 | <0.0001 | <0.0001 | <0.0001 |
| **B** |  | <0.0001 | <0.0001 | <0.0001 | <0.0001 |
| **C** |  |  | 0.9900 | 0.0159 | 0.2431 |
| **D** |  |  |  | 0.0637 | 0.5600 |
| **E** |  |  |  |  | 0.8538 |
| **Phagocytosis** (corresponding to Fig. 4B) | | | | | |
| **Donor** | **B** | **C** | **D** | **E** | **F** |
| **A** | 0.3138 | <0.0001 | <0.0001 | <0.0001 | <0.0001 |
| **B** |  | <0.0001 | <0.0001 | <0.0001 | <0.0001 |
| **C** |  |  | 0.9998 | 0.9996 | 0.9647 |
| **D** |  |  |  | 0.9921 | 0.9943 |
| **E** |  |  |  |  | 0.8760 |
| **Lipid Uptake** (corresponding to Fig. 4C) | | | | | |
| **Donor** | **B** | **C** | **D** | **E** | **F** |
| **A** | >0.9999 | >0.9999 | 0.0430 | >0.9999 | 0.3545 |
| **B** |  | 0.1310 | 0.0012 | 0.1059 | 0.0184 |
| **C** |  |  | >0.9999 | >0.9999 | >0.9999 |
| **D** |  |  |  | >0.9999 | >0.9999 |
| **E** |  |  |  |  | >0.9999 |
| **Inflammasome** (corresponding to Fig. 4D) | | | | | |
| **Donor** | **B** | **C** | **D** | **E** | **F** |
| **A** | >0.9999 | >0.9999 | 0.0184 | 0.7632 | 0.9243 |
| **B** |  | >0.9999 | 0.0839 | >0.9999 | >0.9999 |
| **C** |  |  | 0.4614 | >0.9999 | >0.9999 |
| **D** |  |  |  | >0.9999 | >0.9999 |
| **E** |  |  |  |  | >0.9999 |
| **Mitochondrial Stress** (corresponding to Fig. 4E) | | | | | |
| **Donor** | **B** | **C** | **D** | **E** | **F** |
| **A** | 0.6611 | 0.9417 | 0.1130 | 0.0515 | 0.6918 |
| **B** |  | 0.9969 | 0.8945 | 0.7127 | >0.9999 |
| **C** |  |  | 0.6961 | 0.4899 | 0.9991 |
| **D** |  |  |  | 0.9986 | 0.8029 |
| **E** |  |  |  |  | 0.5745 |

## Supplementary Figures


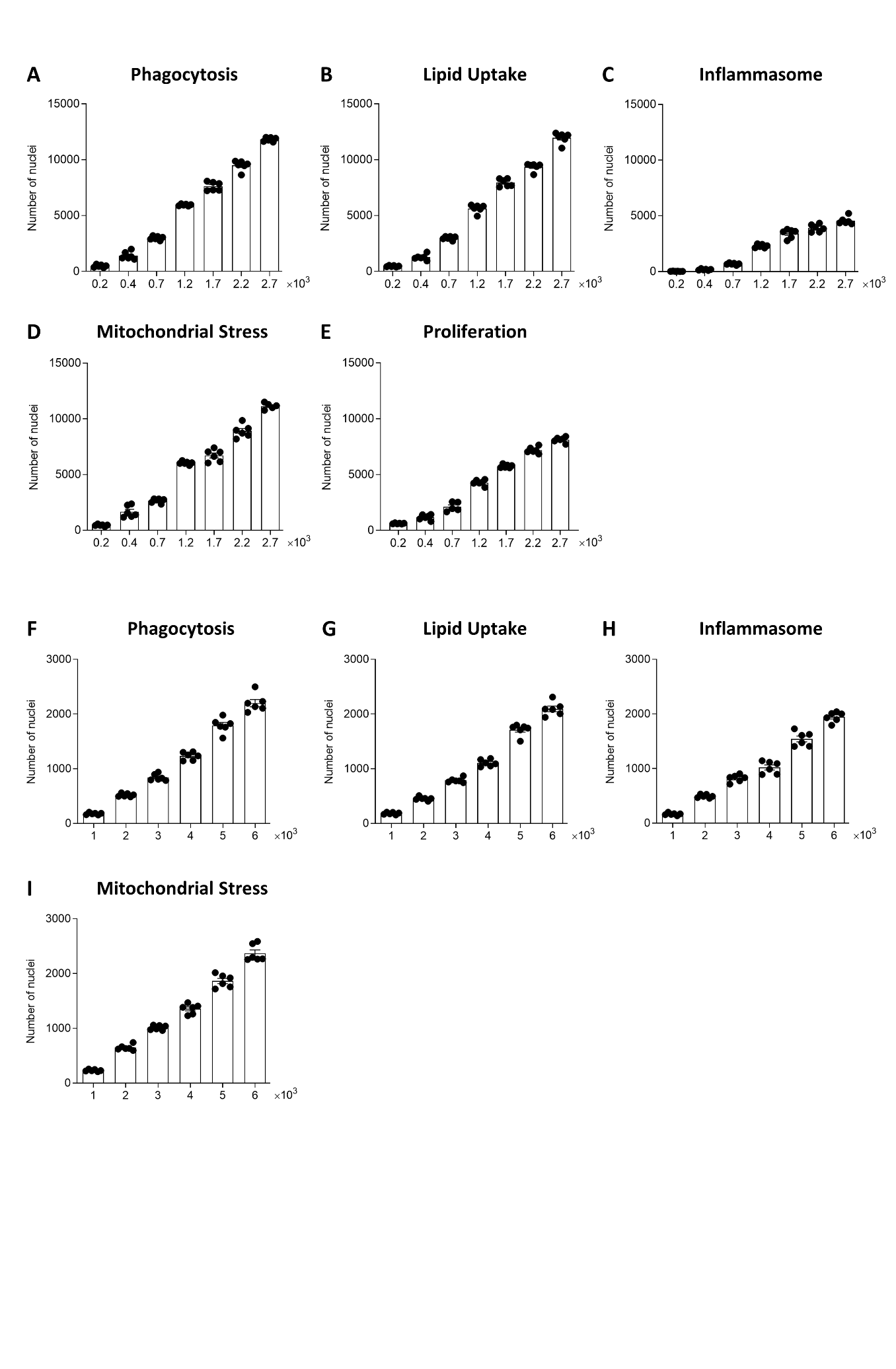


**Supplementary Figure 1.** (A-E) Number of nuclei in functional assays on THP-1 macrophages. (F-I) Number of nuclei in functional assays on primary monocyte-derived macrophages. Density conditions are indicated as × 10^3^ cells/mm^2^.

**
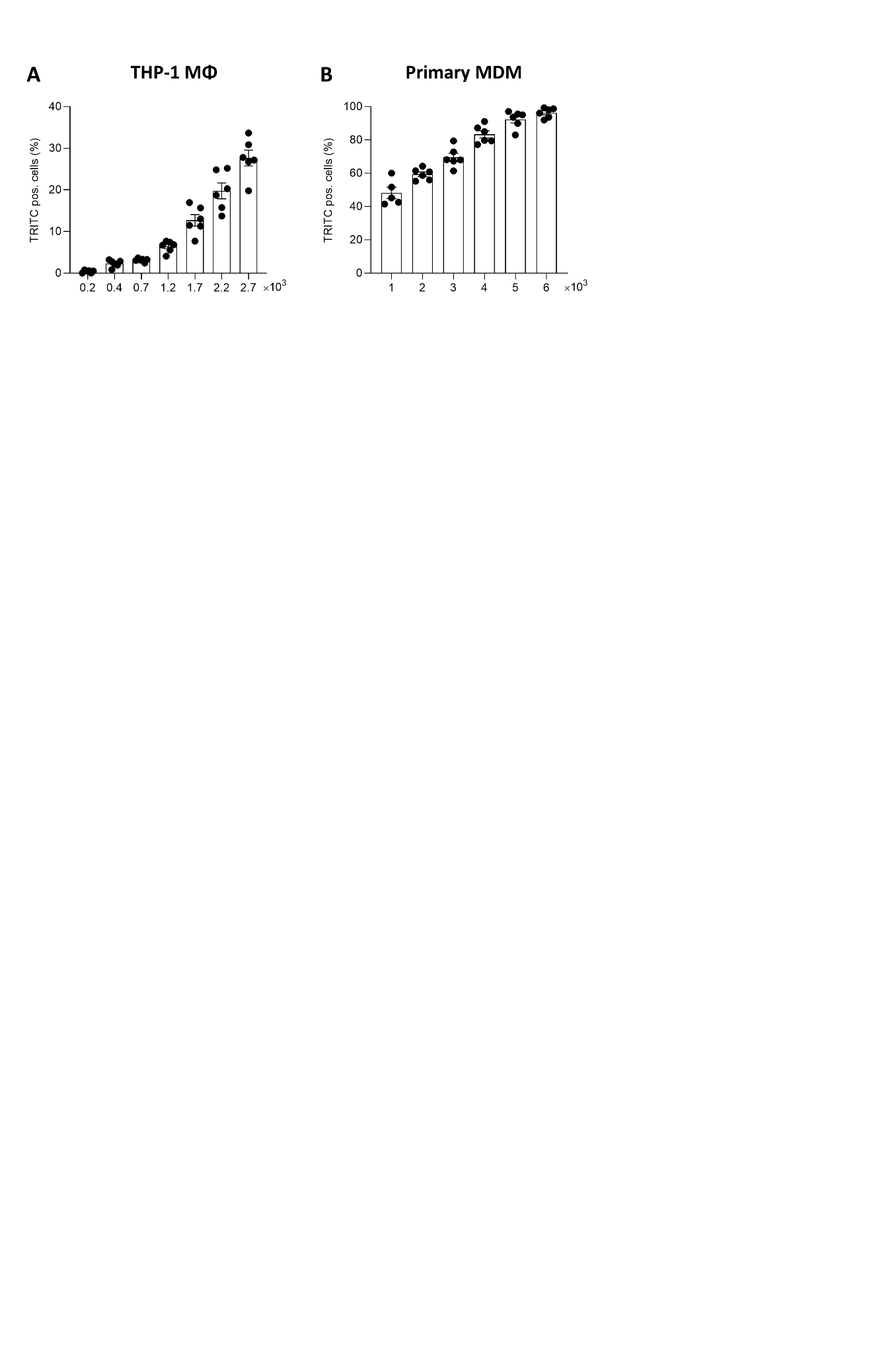
**

**Supplementary Figure 2.** (A) THP-1 cells were seeded at the respective densities (0.2-2.7 x 10^3^ cells/mm²) and differentiated with 2000 nM PMA for 48 hours followed by a resting period of 24 hours. Phagocytic activity was assessed with the MacroScreen platform using an increasing concentration of substrate (zymosan-coated beads) relative to seeding density. (B) CD14-positive monocytes were isolated from PBMCs of 6 healthy donors, pooled and seeded at the respective densities (1-6 x 10^3^ cells/mm²), and differentiated into MΦ using 100 ng/ml macrophage colony-stimulating factor (M-CSF) for 7 days. Phagocytic activity was assessed using a constant ratio of zymosan-coated beads relative to seeding density. Density conditions are indicated as × 10^3^ cells/mm^2^.
